# Plasma Amyloid-Beta Levels in a Pre-Symptomatic Dutch-Type Hereditary Cerebral Amyloid Angiopathy Pedigree: A Cross-Sectional and Longitudinal Investigation

**DOI:** 10.3390/ijms22062931

**Published:** 2021-03-13

**Authors:** Pratishtha Chatterjee, Michelle Tegg, Steve Pedrini, Anne M. Fagan, Chengjie Xiong, Abhay K. Singh, Kevin Taddei, Samantha Gardener, Colin L. Masters, Peter R. Schofield, Gerhard Multhaup, Tammie L. S. Benzinger, John C. Morris, Randall J. Bateman, Steven M. Greenberg, Mark A. van Buchem, Erik Stoops, Hugo Vanderstichele, Charlotte E. Teunissen, Graeme J. Hankey, Marieke J. H. Wermer, Hamid R. Sohrabi, Ralph N. Martins, the Dominantly Inherited Alzheimer Network

**Affiliations:** 1 Department of Biomedical Sciences, Macquarie University, North Ryde, NSW 2109, Australia; pratishtha.chatterjee@mq.edu.au (P.C.); hamid.sohrabi@murdoch.edu.au (H.R.S.); 2 School of Medical and Health Sciences, Edith Cowan University, Joondalup, WA 6027, Australia; m.tegg@ecu.edu.au (M.T.); s.pedrini@ecu.edu.au (S.P.); k.taddei@ecu.edu.au (K.T.); s.gardener@ecu.edu.au (S.G.); 3 Department of Neurology, Washington University, St. Louis, MO 63130, USA; fagana@wustl.edu (A.M.F.); jcmorris@wustl.edu (J.C.M.); batemanr@wustl.edu (R.J.B.); 4 Knight Alzheimer’s Disease Research Center, Washington University, St. Louis, MO 63130, USA; chengjie@wustl.edu (C.X.); benzingert@wustl.edu (T.L.S.B.); 5 Division of Biostatistics, Washington University, St. Louis, MO 63130, USA; 6 Macquarie Business School, Macquarie University, North Ryde, NSW 2109, Australia; abhay.singh@mq.edu.au; 7 Australian Alzheimer’s Research Foundation, Nedlands, WA 6009, Australia; 8 The Florey Institute of Neuroscience and Mental Health, The University of Melbourne, Parkville, VIC 3052, Australia; c.masters@unimelb.edu.au; 9 Neuroscience Research Australia, Sydney, NSW 2031, Australia; p.schofield@neura.edu.au; 10 School of Medical Sciences, University of New South Wales, Sydney, NSW 2052, Australia; 11 Department of Pharmacology and Therapeutics, McGill University, Montreal, QC H3G 1Y6, Canada; gerhard.multhaup@mcgill.ca; 12 Department of Radiology, Washington University School of Medicine, St. Louis, MO 63110, USA; 13 Department of Neurology, Massachusetts General Hospital Stroke Research Center, Harvard Medical School, Boston, MA 02114, USA; sgreenberg@mgh.harvard.edu; 14 Department of Radiology, Leiden University Medical Center, 2333 ZA Leiden, The Netherlands; m.a.van_buchem@lumc.nl; 15 ADx NeuroSciences, 9052 Gent, Belgium; erik.stoops@adxneurosciences.com; 16 Biomarkable, 9000 Gent, Belgium; hugo.vanderstichele@biomarkable.be; 17 Neurochemistry Laboratory, Department of Clinical Chemistry, Amsterdam Neuroscience, Amsterdam University Medical Centers, 1007 MB Amsterdam, The Netherlands; c.teunissen@amsterdamumc.nl; 18 Faculty of Health and Medical Sciences, Medical School, The University of Western Australia, Crawley, WA 6009, Australia; graeme.hankey@uwa.edu.au; 19 Department of Neurology, Leiden University Medical Center, 2333 ZA Leiden, The Netherlands; m.j.h.wermer@lumc.nl; 20 Centre for Healthy Ageing, College of Science, Health, Engineering and Education, Murdoch University, Murdoch, WA 6150, Australia; 21 School of Psychiatry and Clinical Neurosciences, University of Western Australia, Crawley, WA 6009, Australia; 22 The KaRa Institute of Neurological Disease, Macquarie Park, NSW 2113, Australia

**Keywords:** amyloid-beta, plasma amyloid-beta, blood biomarkers, cerebral amyloid angiopathy, early diagnosis, hereditary cerebral haemorrhage with amyloidosis—Dutch type, single molecule array platform

## Abstract

Plasma amyloid-beta (Aβ) has long been investigated as a blood biomarker candidate for Cerebral Amyloid Angiopathy (CAA), however previous findings have been inconsistent which could be attributed to the use of less sensitive assays. This study investigates plasma Aβ alterations between pre-symptomatic Dutch-type hereditary CAA (D-CAA) mutation-carriers (MC) and non-carriers (NC) using two Aβ measurement platforms. Seventeen pre-symptomatic members of a D-CAA pedigree were assembled and followed up 3–4 years later (NC = 8; MC = 9). Plasma Aβ1-40 and Aβ1-42 were cross-sectionally and longitudinally analysed at baseline (T1) and follow-up (T2) and were found to be lower in MCs compared to NCs, cross-sectionally after adjusting for covariates, at both T1(Aβ1-40: *p* = 0.001; Aβ1-42: *p* = 0.0004) and T2 (Aβ1-40: *p* = 0.001; Aβ1-42: *p* = 0.016) employing the Single Molecule Array (Simoa) platform, however no significant differences were observed using the xMAP platform. Further, pairwise longitudinal analyses of plasma Aβ1-40 revealed decreased levels in MCs using data from the Simoa platform (*p* = 0.041) and pairwise longitudinal analyses of plasma Aβ1-42 revealed decreased levels in MCs using data from the xMAP platform (*p* = 0.041). Findings from the Simoa platform suggest that plasma Aβ may add value to a panel of biomarkers for the diagnosis of pre-symptomatic CAA, however, further validation studies in larger sample sets are required.

## 1. Introduction

Sporadic Cerebral Amyloid Angiopathy (CAA) is a leading cause of intracerebral haemorrhage (ICH) and vascular dementia in older adults and is associated with the accumulation of amyloid-β (Aβ) in the cerebral vasculature [1]. The prognosis after CAA-related ICH is poor, with mortality rates reaching as high as 50% or more [2]. CAA pathology is widely seen in patients with Alzheimer’s disease (AD), of whom around 80% exhibit CAA co-morbidity [3]. In addition, the co-presence of CAA in AD, even in the absence of overt ICH, may negatively influence the progression of AD-related neurodegeneration [4,5]. Hence, the ability to diagnose CAA at its earliest stages is crucial.

The Boston criteria aids with the diagnosis of possible and probable CAA [6,7], however, a definitive CAA diagnosis can only be performed by neuropathological examination post-mortem [7]. Early diagnosis using pre-symptomatic CAA markers will provide clinicians with an opportunity to inform patients on the potential risks of thrombolysis or anticoagulation therapy and, treating or lowering other modifiable risk-factors for ICH and vascular dementia such as hypertension, diabetes, hypercholesterolaemia and smoking. 

The Dutch-type hereditary Cerebral Amyloid Angiopathy (D-CAA) also known as hereditary cerebral haemorrhage with amyloidosis Dutch type (HCHWA-D) is a rare autosomal dominant disorder and occurs due to the presence of a guanine to cytosine transversion point mutation on codon 693 of the amyloid precursor protein gene (*APP* E693Q) [8]. The neuropathological hallmark of this mutation is the accumulation of abnormal Aβ (Aβ E22Q) in the cerebral vasculature along with diffuse cerebral plaques, and clinical manifestations of this mutation are recurrent haemorrhagic strokes during mid-life, with dementia usually developing after the first stroke [9,10]. Due to 100% penetrance of the D-CAA mutation if inherited, pre-symptomatic individuals carrying the D-CAA mutation can be identified by genetic analysis, and therefore provide an invaluable human model to study pre-symptomatic biomarkers that may be generalizable to sporadic CAA.

Previous studies evaluated cerebrospinal fluid (CSF) Aβ1-40 and Aβ1-42 and brain Aβ load (via positron emission tomography, PET) as potential CAA markers in D-CAA and sporadic CAA individuals. Lower CSF Aβ1-40 and Aβ1-42 levels and higher PET-Aβ signal were observed in pre-symptomatic and symptomatic D-CAA mutation carrier individuals (MCs) and sporadic CAA individuals compared to D-CAA non-carrier individuals (NCs) and controls, respectively [11,12,13]. However, due to the invasive nature of a lumbar puncture and the costs involved with PET-Aβ imaging, potential blood-based biomarkers for pre-symptomatic CAA need to be investigated [14], as a less invasive and cost-effective measure to detect pre-symptomatic CAA.

Therefore, the aim of the current study was to investigate plasma Aβ1-40 and Aβ1-42 concentrations as potential blood-based biomarkers for CAA. Previous findings have been inconsistent regarding plasma Aβ alterations in CAA [15,16,17], which could be attributed to less sensitive assays employed in the past for the measurement of plasma Aβ1-40 and Aβ1-42. 

To address the previous limitations regarding Aβ assay sensitivity, the current study employed the ultrasensitive Single Molecule Array (Simoa) platform to quantitate and compare plasma Aβ1-40 and Aβ1-42 concentrations in pre-symptomatic MCs and NCs from the same pedigree, cross-sectionally at two timepoints. Additionally, the current study also investigated changes in plasma Aβ1-40 and Aβ1-42 concentrations between MCs and NCs, longitudinally. Further, plasma Aβ1-40 and Aβ1-42 concentrations measured using the Simoa technology platform were compared against observations using the xMAP technology platform, between MCs and NCs. We hypothesised that plasma Aβ1-40 and Aβ1-42 concentrations would be lower in the MCs compared to the NCs cross-sectionally and would decrease in MCs longitudinally.

## 2. Materials and Methods

### 2.1. Participants

Participants were enrolled in the Dominantly Inherited Alzheimer Network (DIAN) study at Edith Cowan University, Western Australia. All participants in the study were from the same *APP* E693Q D-CAA pedigree (*n* = 18) whose ancestors migrated from the Netherlands to Australia. Upon DNA testing, ten participants were found to be positive for the mutation while eight were negative. One MC had a history of stroke and was excluded from the current study in order to utilise a purely pre-symptomatic cohort in the present study. Therefore, eight NCs and nine MCs were included in the current study at baseline (Timepoint 1, T1). Seven NCs and eight MCs from the participants studied at T1 were followed up approximately three (NCs = 6, MCs = 7) to four years (NC = 1, MC = 1) later (Timepoint 2, T2). Blood samples were available for all participants included in the study at T1 and T2, while CSF samples were available for seven NCs and six MCs at T1, and five NCs and three MCs at T2. The study was approved by the human research ethics committees at Macquarie University, Edith Cowan University, Hollywood Private Hospital and Washington University. All participants provided informed written consent. 

### 2.2. Plasma Aβ Measurements and APOE ε4 Genotype Status

Blood processing for plasma collection was performed within 3 h of blood draw, in the morning under fasting conditions, and stored at −80 °C. Plasma Aβ concentrations were measured employing the Amyblood test on the Simoa platform (HDx instrument, Quanterix) using N-terminal-specific monoclonal antibodies provided by ADx NeuroSciences, as described previously [18,19]. Briefly, for Aβ1-40, C-terminal-specific ADx103 (2G3, Aβx-40) was used as the capture antibody and N-terminal-specific ADx101 (3D6, Aβ1-x) was used as the detector antibody. For Aβ1-42, C-terminal-specific ADx102 (21F12, x-42) was used as the capture antibody and N-terminal-specific ADx101 was used as the detector antibody. The percent coefficients of variation (CV) of the three quality control samples ranged between 0–4% and 0–19% for Aβ1-40 and Aβ1-42, respectively. The apolipoprotein E (*APOE*) ε4 genotype status, used as a dichotomous variable (presence/absence), of each study participant was accessed from the DIAN database [20].

### 2.3. Neuropsychological Assessments

Neuropsychological data were obtained from the DIAN database. The Mini Mental State Examination (MMSE) was used as a measure of general cognitive function [21], and the Clinical Dementia Rating (CDR) scale scores, comprising semi-structured interviews for assessing dementia severity [22], were utilised as measures of dementia status within the study.

### 2.4. Assessment of CSF Aβ Concentration and PET Aβ Load

CSF Aβ (Aβ1-40, Aβ1-42) concentrations were obtained from the DIAN database. CSF Aβ1-40 and Aβ1-42 concentrations were measured using INNOTEST β-AMYLOID_(1-40)_ and INNOTEST β-AMYLOID_(1-42)_ (Fujirebio, Ghent, Belgium) as described previously [20]. Brain Aβ load measured using PET coupled with the ligand ^11^C-Pittsburgh Compound B, represented by standard uptake value ratios (SUVR), was accessed from the DIAN database. The SUVR utilised in the current study was calculated with FreeSurfer in the precuneus as the region of interest with the cerebellar grey matter as the reference region, as described previously [23].

### 2.5. Statistical Analysis

Descriptive statistics including means and standard deviations, or proportions were calculated for NCs and MCs, with comparisons employing general linear models or Fisher’s exact test as appropriate. General linear models were employed to compare continuous variables (plasma Aβ1-40 and Aβ1-42) between NCs and MCs, before and after adjustment for the covariates age, sex and *APOE* ε4 carrier status both cross-sectionally (univariate analyses) and longitudinally (repeated measures). Dependent variables were natural log transformed to better approximate normality and variance homogeneity as required. All analyses were carried out using IBM SPSS (Version 26).

## 3. Results

### 3.1. Participant Characteristics

Participant characteristics are presented in Table 1. No significant differences in participant demographics, *APOE* ε4 allele frequency, cognition or history of stroke were present between NCs and pre-symptomatic MCs at T1 and T2.

### 3.2. Cross-Sectional Differences in CSF Aβ and Brain Aβ between D-CAA Mutation Non-Carriers and Carriers

CSF Aβ1-40 and Aβ1-42 concentrations were observed to be significantly lower in the MCs compared to NCs at T1, after adjusting for covariates age, sex and *APOE* ε4 genotype, as expected, and mean CSF Aβ1-40 and Aβ1-42 levels were lower in the MCs compared to the NCs at T2 (statistical analyses were not conducted for CSF Aβ1-40 and Aβ1-42 levels at T2 due to the small CSF sample size at this visit, Table 1). Brain Aβ load assessed via PET was significantly higher in the MCs compared to the NCs at T1 and T2 (Table 1). 

### 3.3. Cross-Sectional Differences in Plasma Aβ between D-CAA Mutation Non-Carriers and Carriers Employing the Ultrasensitive Simoa Technology

**Figure 1:**
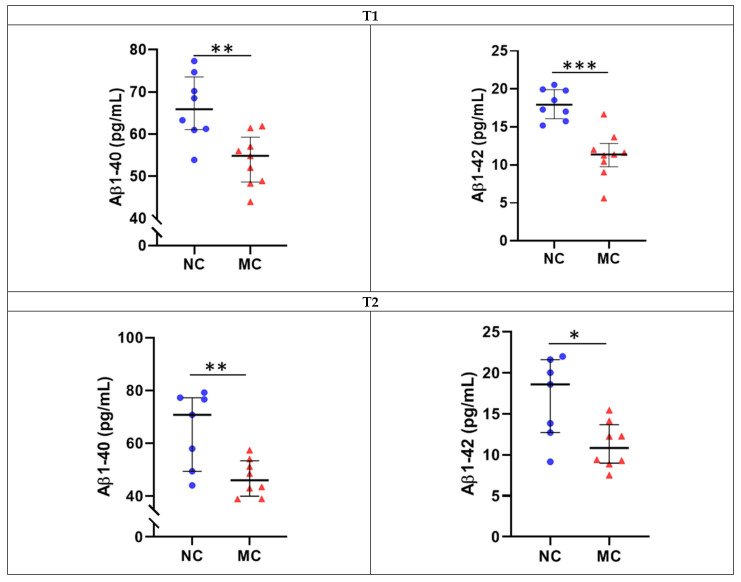
Comparison of plasma Aβ1-40 and Aβ1-42 levels between D-CAA mutation non-carriers and carriers at T1 and T2. Plasma Aβ levels measured using the Amyblood assay on the ultrasensitive Single Molecule Array (Simoa) platform were compared between D-CAA mutation non-carriers (NC) and carriers (MC) at T1 (NC = 8, MC = 9) and T2 (NC = 7, MC = 8, using general linear models. * *p* < 0.05, ** *p* < 0.01, *** *p* < 0.001, adjusted for age, sex and *APOE* ε4 carrier status. *p* < 0.05 was considered significant.

Cross-sectional analyses revealed that plasma Aβ1-40 and Aβ1-42 levels were significantly lower in the MCs compared to the NCs at both timepoints, T1 and T2, before and after adjusting for potential confounding variables, age, sex and *APOE* ε4 genotype (*p* < 0.05, Figure 1, Table 2).

### 3.4. Cross-Sectional Comparison of Plasma Aβ between D-CAA Mutation Non-Carriers and Carriers Employing xMAP Technology

Cross-sectional analyses did not show a significant difference in plasma Aβ1-40 and Aβ1-42 levels between MCs and NCs, after adjusting for covariates age, sex and *APOE* ε4 genotype at both T1 and T2, although mean concentrations of both Aβ1-40 and Aβ1-42 were lower in the MCs compared to the NCs and in line with the ultrasensitive Simoa technology, Aβ1-42 levels were significantly lower in the MCs compared to the NCs at T2 before adjusting for covariates (Supplementary Figure S1, Supplementary Table S1).

### 3.5. Longitudinal Changes in Plasma Aβ Concentrations Employing the Ultrasensitive Simoa Technology

**Figure 2:**
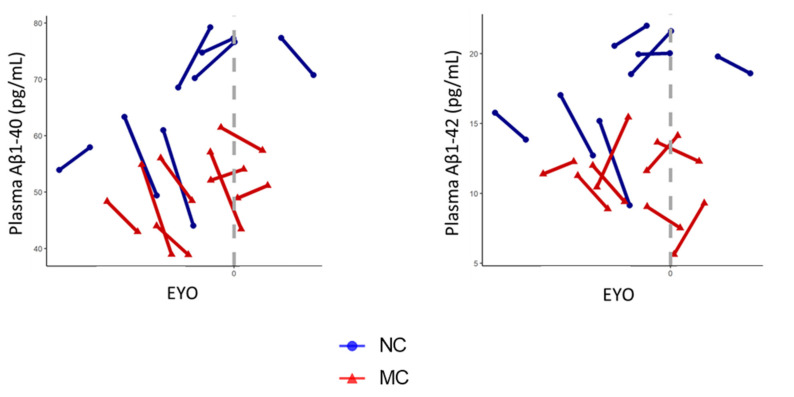
Longitudinal changes in plasma Aβ1-40 and Aβ1-42 levels between D-CAA mutation non-carriers and carriers. Longitudinal measures of plasma Aβ1-40 and Aβ1-42 levels are presented for D-CAA mutation non-carriers (NCs, blue, *n* = 7) and carriers (MCs, red, *n* = 8). Plasma Aβ1-40 and Aβ1-42 levels have been plotted against the expected years to symptom onset (EYO), calculated from the difference of age of the participant and the age at symptom onset of the study pedigree.

Longitudinal analyses revealed that Aβ1-40 concentrations decreased over time (T1 to T2) with a trend towards significance (*p* = 0.057), before adjusting for covariates, age, sex and *APOE* ε4 genotype, wherein pairwise comparisons showed significantly decreased mean plasma Aβ1-40 concentrations in MCs (*p* = 0.045, Table 3). After adjusting for covariates, age, sex and *APOE* ε4 genotype, longitudinal analyses revealed that Aβ1-40 concentrations significantly decreased over time (T1 to T2, *p* = 0.028), wherein a pairwise comparison showed significantly decreased mean plasma Aβ1-40 concentrations in MCs (*p* = 0.041, Table 3). No statistically significant effect of time on plasma Aβ1-40 in NCs was observed before or after adjusting for covariates (Table 3). Time * Mutation status interaction revealed no significant differences in plasma Aβ1-40 changes between MCs and NCs, either before or after adjusting for potential confounding variables age, sex and *APOE* ε4 genotype (Figure 2, Table 3) which could be attributed to the modest sample size.

No statistically significant effect on plasma Aβ1-42 concentrations was observed over time (T1 to T2), including pairwise comparisons in MCs or NCs, or Time * Mutation status interactions, before and after adjusting for covariates, age, sex and *APOE* ε4 genotype (Table 3).

### 3.6. Longitudinal Changes in Plasma Aβ Concentrations Employing the xMAP Technology

No statistically significant effect on plasma Aβ1-40 concentrations were observed over time (T1 to T2), including pairwise comparisons in MCs or NCs, and Time * Mutation status interactions, before and after adjusting for covariates, age, sex and *APOE* ε4 genotype (Supplementary Table S2).

Longitudinal analyses revealed that Aβ1-42 concentrations decreased over time (T1 to T2) with a trend towards significance, before (*p* = 0.066) and after (*p* = 0.084) adjusting for covariates, age, sex and *APOE* ε4 genotype, wherein pairwise comparisons showed significantly decreased mean plasma Aβ1-42 concentrations in MCs (*p* < 0.05, Supplementary Table S2). No statistically significant effect of time on plasma Aβ1-42 in NCs was observed before or after adjusting for covariates. Time * Mutation status interaction revealed no significant differences in plasma Aβ1-42 changes between MCs and NCs, either before or after adjusting for potential confounding variables age, sex and *APOE* ε4 genotype (Supplementary Table S2).

## 4. Discussion

The current study is the first to report on plasma Aβ1-40 and Aβ1-42 alterations between pre-symptomatic D-CAA MCs (i.e., prior to stroke and cognitive impairment) and NCs from the same pedigree, using the ultrasensitive Simoa technology, wherein lower plasma Aβ1-40 and Aβ1-42 levels were observed in MCs compared to the NCs in line with our hypothesis. These observations were consistent at both study timepoints which were at least three years apart. Findings from these cross-sectional analyses using the ultrasensitive Simoa technology platform suggest that plasma Aβ1-40 and Aβ1-42 may serve as potential candidates for a CAA diagnostic biomarker panel, although further validation studies are necessary in sporadic CAA. 

While our cross-sectional observations of lower plasma Aβ1-40 and Aβ1-42 in MCs compared to NCs within the current study were consistent at two independent timepoints using the ultrasensitive Simoa technology platform, previous studies investigating plasma Aβ1-40 and Aβ1-42 in CAA (D-CAA and sporadic-CAA) have been inconsistent. One study reported no significant difference in plasma Aβ1-40 levels but significantly lower plasma Aβ1-42 levels in D-CAA MCs compared to NCs [16], while a study on sporadic CAA individuals comprising 80% probable CAA and 20% definite CAA, reported no significant difference in plasma Aβ1-40 and Aβ1-42 concentrations compared to controls [15], and yet another study reported significantly higher plasma Aβ1-40 and Aβ1-42 in probable CAA compared to controls [17]. These contrasting observations could be attributed to the sensitivity of the assays employed to measure plasma Aβ concentrations. For example, plasma Aβ1-40 and Aβ1-42 concentrations measured on the xMAP platform, as employed by Hernandez-Guillamon and colleagues [17], when used in participants within the present study (at both timepoints, T1 and T2), also did not appear to be significantly different cross-sectionally after adjusting for covariates (Supplementary Figure S1, Supplementary Table S1), therefore highlighting the differences between assay platforms. However, it is also important to note that participants in the present study had no history of stroke and no cognitive impairment, as opposed to participants from the aforementioned studies, which may have contributed to the differences in our observations, since stroke related cognitive impairment may be associated with changes in Aβ levels [24]. 

The lower levels of plasma Aβ1-40 and Aβ1-42 and higher brain Aβ load observed in D-CAA mutation carriers have been posited to be attributed to altered processing and transport of Aβ, wherein the D-CAA mutation impairs Aβ elimination from the brain by reducing transport across the blood–brain barrier (BBB) and the vascular drainage pathways [16,25]. CAA patients have been previously shown to have higher microvascular expression of the receptor for advanced glycation end products (RAGE) and reduced low-density lipoprotein receptor-related protein 1 (LRP1) expression, which regulate the influx and efflux, respectively, of Aβ across the BBB [26,27]. Therefore, the results of this study are consistent with previous literature that has demonstrated impairment of Aβ transport mechanisms between brain and periphery in CAA. Additionally, the D-CAA MCs show diffuse-type plaque in cerebral cortex, and therefore lower plasma Aβ1-42 levels in the MCs could be affected by the status of cerebral beta amyloidosis [28].

Longitudinal changes in mean plasma Aβ1-40 and Aβ1-42 concentrations, measured using the Simoa and xMAP platforms, respectively, were observed to significantly decrease with time in MCs, but not in NCs. These observations may reflect increased D-CAA pathophysiology with time, i.e., further vascular or cerebral Aβ deposition with time in D-CAA cases. However, a larger sample size with more datapoints is required to confirm this. Furthermore, observations of significantly declining plasma Aβ1-40 in MCs employing the Simoa platform and significantly declining plasma Aβ1-42 in MCs employing the xMAP platform, suggest that platform- and/or antibody- or isoform-specific differences might exist, however, further validation studies are required. 

A limitation within the current study is its modest sample size. However, the current study has several strengths including the utilisation of NC and MC plasma samples from the same D-CAA pedigree, the validation of lower plasma Aβ1-40 and Aβ1-42 concentrations in D-CAA MCs cross-sectionally at two timepoints (T1 and T2), and the employment of an ultrasensitive platform for the measurement of plasma Aβ1-40 and Aβ1-42 concentrations.

An important consideration from the findings of this study would be, whether observations from the Dutch-CAA cases will be applicable to sporadic CAA cases, in the context of a biomarker, within the presymptomatic phase. Sporadic CAA may be more heterogeneous, wherein faulty vascular/perivascular clearance may be more important than overproduction of Aβ in some sporadic CAA. Faulty clearance mechanism in sporadic CAA may be more vulnerable to vascular risk factors such as hypertension or diabetes. Additionally, given that clinically most sporadic CAA coexist with AD, it is important to note that the use of a blood Aβ assay alone is likely to be limited, while a panel of biomarkers (including Aβ) is more likely to have success, for the diagnosis of pre-symptomatic CAA.

To conclude, cross-sectional analyses of data obtained from the ultrasensitive Simoa platform revealed that plasma Aβ1-40 and Aβ1-42 concentrations are lower in pre-symptomatic D-CAA MCs compared to NCs and longitudinal analyses revealed that plasma Aβ1-40 levels decreased in MC. Additionally, longitudinal analyses of data obtained from the xMAP platform revealed that plasma Aβ1-42 levels are decreased in MCs. While platform differences might exist, findings from this study suggest that plasma Aβ may add value to a panel of biomarkers for the diagnosis of pre-symptomatic CAA, however, further validation studies in larger samples sets are required and future validation studies are necessary in sporadic-CAA. 

## Figures and Tables

**Table 1:** Participant characteristics at T1 and T2. Characteristics including sex, age, expected years to symptom onset (EYO) based on age at symptom onset of biological mutation carrier parent, *APOE* ε4 status, Mini-Mental State Examination (MMSE) scores, Clinical Dementia Rating (CDR) scale scores, history of stroke, cerebrospinal fluid (CSF) Aβ1-40 levels, CSF Aβ1-42 levels and brain Aβ load signal have been compared between Dutch-type hereditary cerebral amyloid angiopathy (D-CAA) mutation non-carriers (NCs) and pre-symptomatic carriers (MCs) from the same pedigree at T1 and T2. Fisher’s exact test or linear models were employed as appropriate. CSF Aβ1-42 and Aβ1-40 were measured using the INNOTEST β-AMYLOID(1-40) and INNOTEST β-AMYLOID(1-42) ELISA (Fujirebio, Ghent, Belgium). Brain Aβ load was measured via positron emission tomography and is presented as the standard uptake value ratio (SUVR) of the ligand, ^11^C Pittsburgh Compound B, uptake in the precuneus as the region of interest, with the cerebellar grey matter as the reference region. *p* ^a^ represents *p*-values adjusted for age, sex and *APOE* ε4 carrier status. *p* < 0.05 was considered significant and are presented in bold font.

	D-CAA NC	D-CAA MC	*p*	*p* ^a^
**T1**				
N	8	9	-	-
Sex (M/F)	3/5	3/6	1.00	-
Age (Mean ± SD)	43.5 ± 6.57	44.11 ± 4.31	0.822	-
EYO (years; Mean ± SD)	−6.09 ± 6.43	−5.27 ± 4.30	0.759	-
*APOE* ε4 (%)	37.5	22.2	0.620	-
MMSE (Mean ± SD)	28.63 ± 1.60	28.67 ± 1.41	0.955	-
CDR global = 0 (%)	100	100	-	-
Stroke (%)	0	0	-	-
CSF Aβ1-40 (pg/mL; Mean ± SD) ^#^	9882.92 ± 3076.97	6971.35 ± 1088.65	0.051	**0.039**
CSF Aβ1-42 (pg/mL; Mean ± SD) ^#^	618.63 ± 206.81	311.67 ± 154.85	**0.012**	**0.007**
Brain Aβ load (SUVR, Mean ± SD)	0.994 ± 0.064	1.268 ± 0.248	**0.009**	**0.011**
**T2**				
N	7	8	-	-
Sex (M/F)	2/5	2/6	1.00	-
Age (Mean ± SD)	46.57 ± 7.21	46.25 ± 4.65	0.830	-
EYO (years; Mean ± SD)	−2.97 ± 7.02	−2.12 ± 4.75	0.785	-
*APOE* ε4 (%)	42.86	25	0.608	-
MMSE (Mean ± SD)	28.71 ± 1.38	28.63 ± 0.92	0.883	-
CDR global = 0 (%)	100	100	-	-
Stroke (%)	0	0	-	-
CSF Aβ1-40 (pg/mL; Mean ± SD) ^#^	7483.03 ± 1454.50	3933.53 ± 1993.14	-	-
CSF Aβ1-42 (pg/mL; Mean ± SD) ^#^	1078.44 ± 162.86	346.61 ± 147.11	-	-
Brain Aβ load (SUVR, Mean ± SD)	1.06 ± 0.034	1.35 ± 0.25	**0.009**	**0.001**

^#^ CSF parameters are only presented for seven NCs and six MCs at T1, and for three NCs and five MCs at T2, due to CSF sample unavailability. Statistical comparisons of CSF parameters between NCs and MCs were not conducted for T2 due to the small sample size.

**Table 2:** Cross-sectional comparison of plasma Aβ levels between pre-symptomatic D-CAA mutation carriers and non-carriers measured on the ultrasensitive Simoa platform. Plasma Aβ levels were compared between D-CAA mutation carriers and non-carriers using general linear models separately at T1 and T2. Data are presented at T1 and T2. Data are presented as Mean ± SD in pg/mL. *p* ^a^ represents p-values adjusted for age, sex and *APOE* ε4 carrier status. ⱡ represents *p*-values obtained from natural log transformed Aβ1-40 or Aβ1-42 concentrations to better approximate normality. *p*-values < 0.05 were considered significant and are presented in bold font.

	D-CAA NC	D-CAA MC	*p*	*p* ^a^
**T1**	***n*** **= 8**	***n*** **= 9**		
Aβ1-40	66.30 ± 7.83	53.87 ± 6.07	**0.002**	**0.001**
Aβ1-42	18.02 ± 2.01	11.31 ± 3.02	**0.00009**	**0.0004**
**T2**	***n*** **= 7**	***n*** **= 8**		
Aβ1-40	65.04 ± 14.45	46.92 ± 6.93	**0.008 ^ⱡ^**	**0.001 ^ⱡ^**
Aβ1-42	16.85 ± 4.96	11.16 ± 2.79	**0.020 ^ⱡ^**	**0.016**

**Table 3:** Longitudinal comparison of plasma Aβ levels between pre-symptomatic D-CAA mutation carriers and non-carriers. Plasma Aβ levels were measured on the Simoa platform and were compared between D-CAA mutation carriers (MC) and non-carriers (NC) using repeated measures analyses at T1 and T2, before and after adjustment for covariates age, sex and *APOE* ε4 carrier status, using a factorial design (all two-way interactions with time included). Data are presented as Mean ± SD in pg/mL. ⱡ represents *p*-values obtained from natural log transformed Aβ1-40 or Aβ1-42 concentrations to better approximate normality. * *p*-values < 0.05 were considered significant and are presented in bold font.

	D-CAA NC (*n* = 7)		D-CAA MC (*n* = 8)		Time			Time (Adjusted for Covariates)			Time * Mutation	Time * Mutation (Adjusted for Covariates)
	T1	T2	T1	T2	*p*	Pairwise		*p*	Pairwise		*p*	*p*
						*p* ^NC^	*p* ^MC^		*p* ^NC^	*p* ^MC^		
Aβ1-40	67.01 ± 8.17	65.04 ± 14.45	52.86 ± 5.62	46.92 ± 6.93	0.057 ^ⱡ^	0.449 ^ⱡ^	**0.045** ^ⱡ^	**0.028** ^ⱡ^	0.227 ^ⱡ^	**0.041** ^ⱡ^	0.362 ^ⱡ^	0.526 ^ⱡ^
Aβ1-42	18.12 ± 2.14	16.85 ± 4.96	10.63 ± 2.41	11.16 ± 2.79	0.665 ^ⱡ^	0.290 ^ⱡ^	0.605 ^ⱡ^	0.584	0.159	0.436	0.264 ^ⱡ^	0.130

## Data Availability

The data presented in this study are available on request from the corresponding author.

## Supplementary Materials

The following are available online at https://www.mdpi.com/1422-0067/22/6/2931/s1.
