# Supplementary material for: Plasma Amyloid-Beta Levels in a Pre-Symptomatic Dutch-Type Hereditary Cerebral Amyloid Angiopathy Pedigree: A Cross-Sectional and Longitudinal Investigation
Source: Int J Mol Sci. 2021 Mar 13;22(6):2931. doi: 10.3390/ijms22062931 (PMC8000178; doi:10.3390/ijms22062931)
Supplement: Supplementary file 1 [file ijms-22-02931-s001.pdf]

Supplementary Figure 1. Comparison of plasma Aβ1-40 and Aβ1-42 levels A.) cross-sectionally between D-CAA mutation non-carriers and carriers at T1 and T2 utilising the xMAP INNO-BIA Plasma Aβ Forms Multiplex Assay (Innogenetics, Ghent, Belgium) and B.) longitudinally between D-CAA mutation non-carriers and carriers.

A.)

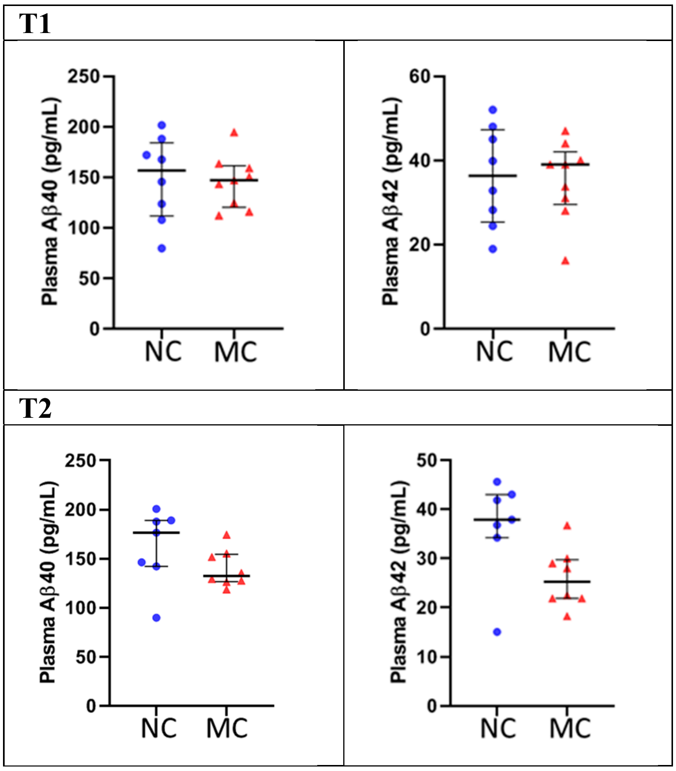

B.)

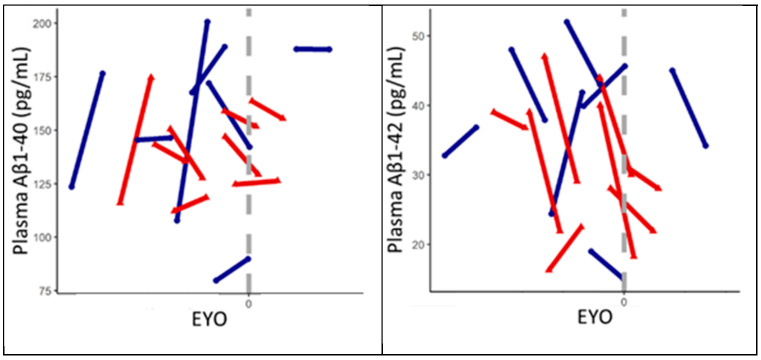

Supplementary Table 1. Cross-sectional comparison of plasma Aβ levels between pre-symptomatic D-CAA mutation carriers and non-carriers. Plasma Aβ levels were measured utilising the xMAP INNO-BIA Plasma Aβ Forms Multiplex Assay (Innogenetics, Ghent, Belgium) and were compared between D-CAA mutation carriers and non-carriers using general linear models separately at T1 and T2. Data are presented as Mean±SD in pg/mL. p[a] represents p-values adjusted for age, sex and *APOE* ε4 carrier status. p<.05 was considered significant and is presented in bold font.

	**D-CAA NC**	**D-CAA MC**	**p**	**p[a]**
**T1**	**n=8**	**n=9**		
Aβ1-40	148.23±41.93	145.66±25.93	.880	.784
Aβ1-42	36.16±11.90	35.35±9.33	.877	.780
**T2**	**n=7**	**n=8**		
Aβ1-40	161.79±38.59	139.89±18.91	.177	.180
Aβ1-42	36.35±10.18	26.05±5.98	**.030**	.064

Supplementary Table 2. Longitudinal comparison of plasma Aβ1-40 and Aβ1-42 measured by xMAP INNO-BIA Plasma Aβ Forms Multiplex Assay (Innogenetics, Ghent, Belgium) between pre-symptomatic D-CAA mutation carriers and non-carriers. Plasma Aβ1-40 and Aβ1-42 concentrations measured using the xMAP INNO-BIA Plasma Aβ Forms Multiplex Assay platform were compared between D-CAA mutation carriers (MC) and non-carriers (NC) using repeated measures analyses at T1 and T2, before and after adjustment for covariates age, sex and *APOE* ε4 carrier status, using a factorial design (all two-way interactions with time included). Data are presented as Mean±SD in pg/mL. $p<.05$ was considered significant and is presented in bold font.

	**D-CAA NC (n=7)**		**D-CAA MC (n=8)**		**Time**			**Time (adjusted for covariates)**			**Time*Mutation**	**Time*Mutation (adjusted for covariates)**
	**T1**	**T2**	**T1**	**T2**	***p***	*Pairwise* p^{NC}	*Pairwise* p^{MC}	***p***	*Pairwise* p^{NC}	*Pairwise* p^{MC}	***p***	***p***
Aβ1-40	140.61±38.85	161.78±38.59	139.52±19.52	139.88±18.91	.231	.114	.976	.225	.115	.976	.246	.248
Aβ1-42	37.30±12.37	36.35±10.18	35.55±9.95	26.05±5.98	.066	.807	**.019**	.084	.687	**.041**	.124	.229
